# The contribution of high frequencies to human brain activity underlying horizontal localization of natural spatial sounds

**DOI:** 10.1186/1471-2202-8-78

**Published:** 2007-09-26

**Authors:** Sakari Leino, Patrick JC May, Paavo Alku, Lassi A Liikkanen, Hannu Tiitinen

**Affiliations:** 1 Apperception & Cortical Dynamics (ACD), Department of Psychology, University of Helsinki, Finland; 2 BioMag Laboratory, Hospital District of Helsinki and Uusimaa HUSLAB, Helsinki University Central Hospital, Finland; 3 Laboratory of Acoustics and Audio Signal Processing, Helsinki University of Technology, Finland

## Abstract

**Background:**

In the field of auditory neuroscience, much research has focused on the neural processes underlying human sound localization. A recent magnetoencephalography (MEG) study investigated localization-related brain activity by measuring the N1m event-related response originating in the auditory cortex. It was found that the dynamic range of the right-hemispheric N1m response, defined as the mean difference in response magnitude between contralateral and ipsilateral stimulation, reflects cortical activity related to the discrimination of horizontal sound direction. Interestingly, the results also suggested that the presence of realistic spectral information within horizontally located spatial sounds resulted in a larger right-hemispheric N1m dynamic range. Spectral cues being predominant at high frequencies, the present study further investigated the issue by removing frequencies from the spatial stimuli with low-pass filtering. This resulted in a stepwise elimination of direction-specific spectral information. Interaural time and level differences were kept constant. The original, unfiltered stimuli were broadband noise signals presented from five frontal horizontal directions and binaurally recorded for eight human subjects with miniature microphones placed in each subject's ear canals. Stimuli were presented to the subjects during MEG registration and in a behavioral listening experiment.

**Results:**

The dynamic range of the right-hemispheric N1m amplitude was not significantly affected even when all frequencies above 600 Hz were removed. The dynamic range of the left-hemispheric N1m response was significantly diminished by the removal of frequencies over 7.5 kHz. The subjects' behavioral sound direction discrimination was only affected by the removal of frequencies over 600 Hz.

**Conclusion:**

In accord with previous psychophysical findings, the current results indicate that frontal horizontal sound localization and related right-hemispheric cortical processes are insensitive to the presence of high-frequency spectral information. The previously described changes in localization-related brain activity, reflected in the enlarged N1m dynamic range elicited by natural spatial stimuli, can most likely be attributed to the processing of individualized spatial cues present already at relatively low frequencies. The left-hemispheric effect could be an indication of left-hemispheric processing of high-frequency sound information unrelated to sound localization. Taken together, these results provide converging evidence for a hemispheric asymmetry in sound localization.

## Background

In the field of auditory neuroscience, an enduring topic of interest is the study of the neural processes underlying the perception of spatial sound properties and the localization of sound sources in the three-dimensional environment. Stimuli with spatial properties have been used to study sound localization with both psychophysical and neuroscientific measures. A century of psychophysical research has so far meticulously documented the psychoacoustic and behavioral aspects of sound localization (for reviews, see [1,2]). The neural basis of sound localization has also been extensively studied within animal models (see e.g., [3,4]) and neuropsychology (see e.g., [5-7]). More recently, functional neuroimaging methods have been utilized to study human sound localization. Such methods have included electrophysiological (e.g., [8-11]), neuromagnetic (e.g., [12-19]) and hemodynamic (e.g., [20-23]) measurements.

When performing localization of spatial sounds, the human auditory system can utilize several types of auditory cues, and previous brain measurements have suggested that the different cues are processed by different cortical mechanisms (see e.g., [9-11,13,24]). Interaural time differences (ITDs), caused by a differential distance between the sound source and the ears, and interaural level differences (ILDs), caused by the acoustical shadowing effects of the head, are analyzed by the auditory system to determine the horizontal direction of the sound source. ITD cues usually dominate when low-frequency sound information (below approximately 1500 Hz) is present, whereas ILD cues are used predominantly at higher frequencies [1,2,25]. In addition to the interaural differences, any exterior sound signal entering the auditory system is exposed to the filtering effects of the pinnae, the head and the body, causing modulations of the sound spectrum that vary consistently over sound direction. Spectral localization cues are usually predominant at high frequencies above approximately 6 kHz, although spectral modifications at much lower frequencies can also contribute to sound localization [1,2,26,27].

Spectral localization cues are generally thought to be utilized primarily in the discrimination of differences in elevation (where interaural differences are not useful) and to be less important in horizontal localization [1,2]. However, spectral cues have also been shown to contribute to discrimination between front and back directions [28], and a study of chronic monaural sound localization has suggested that some monaurally deaf listeners can utilize spectral cues for horizontal localization as well [29]. Moreover, in a striking contrast to sounds with interaural cues only, spatial sounds containing natural binaural spectral information are subjectively perceived as originating outside of the listener's head (see e.g., [1,30]). In other words, some processing of spectral cues must take place in order for the listener to localize a sound source as lying outside of the head in the first place. Taken together, interaural and spectral localization cues are analyzed by distinct and, to some extent, independent processes, with the analysis of interaural cues dominating horizontal localization and spectral cues contributing most prominently to vertical localization. The exact nature of the interaction and final integration between the different cues is still unclear (see e.g., [10]). It has been suggested, for example, that in both horizontal and vertical localization, ITD cues are used to establish the locus of possible source directions, and the analysis of ILD and spectral cues is then utilized to resolve possible confusions [25].

So far, many studies on the neural basis of sound localization have utilized sounds containing ITD and ILD cues as stimulus material. However, as described above, interaural differences only represent a subset of all natural sound localization cues. Loudspeakers can be used to generate genuine spatial sounds, but they cannot be used in magnetoencephalography (MEG) or functional magnetic resonance imaging (fMRI) measurements due to their magnetic interference. However, spatial stimuli presentable through non-magnetic earphones, and thus eligible for MEG and fMRI experiments, can be constructed using head-related transfer functions (HRTFs) [31], which simulate the spectral filtering effects of the pinnae, the head, and the body. Generic (average) HRTFs can be used, the transfer functions can be derived from dummy-head measurements or, for more realistic spatial stimuli, individualized HRTFs can be measured (e.g., [12,13,15-17,21]). Individualized, realistic spatial stimuli of very high quality can also be generated by separately recording each sound, sequentially presented through several loudspeakers, with binaural miniature microphones placed inside the ear canals of the subject [17,19].

Many previous studies have found an interesting hemispherical asymmetry in human perception and localization of spatial sounds, indicating a dominant role for right-hemispheric auditory areas (e.g., [6,8,14-17,23,32]). In particular, Palomäki et al. [17] recently conducted an MEG experiment to further investigate the relationship between the discrimination of sound location and the pronounced activity of the auditory areas of the right hemisphere. These authors investigated the dynamics and angular organization of the auditory N1m response, a prominent event-related magnetic brain response originating in the auditory cortex and peaking at approximately 100 ms after stimulus onset. More specifically, they scrutinized the differences in response amplitude between contralateral hemifield and ipsilateral hemifield stimulation for both hemispheres, referring to the average contra-ipsi difference as the dynamic range of the N1m amplitude. Importantly, a larger dynamic range of the right-hemispheric N1m was linked to better accuracy in horizontal direction discrimination, measured in a behavioral listening experiment. Furthermore, individualized, binaurally recorded broadband sound signals containing realistic interaural and spectral cues elicited a right-hemispheric N1m with a larger dynamic range than broadband sounds containing isolated interaural cues only. The larger dynamic range was suggested to reflect the greater availability of localization cues within the natural, binaurally recorded stimuli, and the right-hemispheric N1m dynamic range in general was suggested to reflect how realistic and discriminable spatial sounds are.

In the experiment of Palomäki et al. [17], the binaurally recorded natural sound stimuli most profoundly differed from the stimuli containing isolated interaural cues with respect to their spectral structure. All of the stimuli with isolated ITD and ILD cues had the same non-individualized, binaurally equivalent spectral structure, corresponding to the grand-averaged spectra of those binaurally recorded stimuli which originated from directly in front of the subjects. The spectral structure thus remained the same regardless of the perceived horizontal direction of the stimulus. The differences in perceived direction were achieved solely by interaural cues, constructed to closely reflect the interaural cues present in the binaurally recorded stimuli but ultimately resulting in an internalized perception. In contrast, the spectra of the binaurally recorded stimuli, perceived as originating outside of the head, contained fully realistic direction- and ear-specific modulations caused by each individual's pinnae, head and body. This gives rise to the interesting interpretation that the larger dynamic range of the N1m response, linked in the previous study to better horizontal localization accuracy, reflected the presence of natural, individualized spectral information. While the discrimination of a sound's direction of origin in the horizontal plane is generally dominated by ITD and ILD cues [1,2], it does appear that externalized perception of any auditory object, regardless of the direction, is likely to entail some processing of spectral cues as well. The availability of natural and binaural spectral localization cues within the horizontally located sounds may thus conceivably have contributed to the brain activity related to their perception and localization.

The present study, comprising MEG measurements and subsequent behavioral sound direction discrimination experiments, was conducted to further investigate the possible contribution of spectral information to the changes in localization-related brain activity reported in the previous study [17]. The same individualized stimuli as in the previous study were utilized also in the present experiment. Stimuli from five frontal horizontal directions containing binaural direction-dependent spectral structures and interaural time and level differences were used. Exclusively frontal directions were chosen to avoid any effects related to the contribution of spectral cues to front/back discrimination (cf. [28]). To manipulate the spectral structure of the stimuli, the original 10 kHz broadband signals were low-pass filtered in a stepwise manner (with cut-off frequencies at 7.5 kHz, 3 kHz, and 600 Hz) while carefully preserving any interaural differences. As spectral localization cues occur predominantly at high frequencies, any contribution by spectral information to localization-related cortical activity was expected to be revealed by the reduction in bandwidth. Specifically, should the increased brain activity reported previously have been due to the presence of natural, high-frequency spectral information, the dynamic range of the right-hemispheric N1m response in the present experiment was expected to be diminished as a result of the removal of high-frequency information. Conversely, should the dynamic range of the N1m response remain unaffected in the present experiment, the result would indicate that the neural processes indexed by the N1m dynamic range are insensitive to the availability of high-frequency spectral localization cues, and that the previously reported larger dynamic range in the case of natural stimuli was most likely due to individualized information present at lower frequencies.

## Results

**Figure 1:**
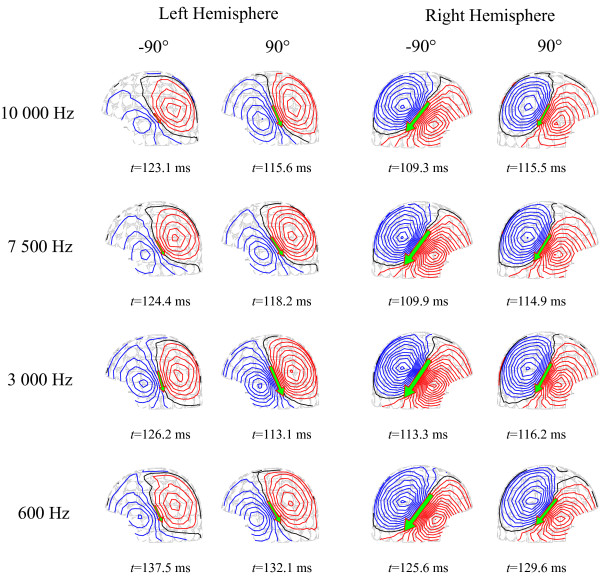
**Representative N1m responses**. Equivalent current dipoles (ECDs) calculated for the grand-averaged N1m response at the average peak latency *t*. The figure shows ECDs for stimuli with different bandwidths originating from directions -90° and 90°, calculated separately for the left and right hemisphere (contour step 20 fT/cm).

**Figure 2:**
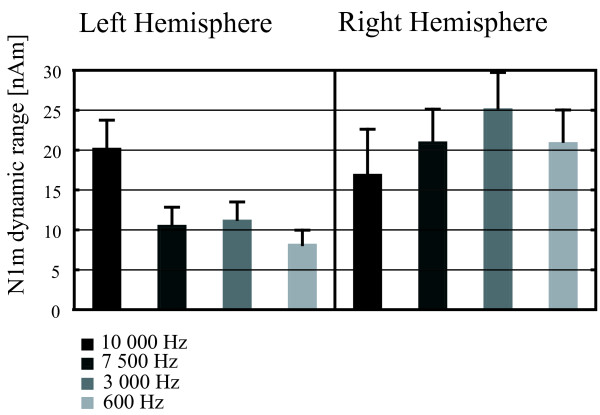
**N1m dynamic range**. The grand-average dynamic range of the N1m amplitude for stimuli with different bandwidths. For the analysis of the dynamic range within each hemisphere, the N1m amplitudes for ipsilateral stimuli were subtracted from their contralateral counterparts. The left-hemispheric dynamic range was diminished by the reduction in bandwidth, while its right-hemispheric counterpart was not significantly affected. Error bars indicate SEM.

All stimuli elicited prominent bilateral N1m responses, with equivalent current dipole (ECD) modeling indicating sources in the vicinity of the auditory cortices of both hemispheres. Fig. 1 shows representative ECDs calculated for the N1m responses, and Table 1 summarizes the average N1m amplitudes and peak latencies in the left and right hemispheres for the four bandwidths used. As depicted in Fig. 2, the dynamic range of the right-hemispheric N1m amplitude was not significantly affected by changes in the bandwidth, while the dynamic range of the left-hemispheric N1m amplitude was smaller when stimuli with any of the narrower bandwidths were presented (*F*[3,18] = 3.66, *p *< 0.05).

**Table 1:** N1m amplitudes and latencies

		Left hemisphere		Right hemisphere	
Bandwidth	Direction	Latency [ms]	Amplitude [nAm]	Latency [ms]	Amplitude [nAm]

**10 000 Hz**	**-90°**	**123,1**	**18,7**	**109,3**	**29,5**
	-45°	120,4	17,2	114,0	28,1
	0°	113,7	20,3	105,5	30,3
	45°	123,9	24,5	117,4	22,5
	90°	115,6	23,2	115,5	20,2
**7 500 Hz**	**-90°**	**124,4**	**20,8**	**109,9**	**33,7**
	-45°	120,4	18,7	113,6	32,6
	0°	116,6	20,9	112,4	30,4
	45°	120,2	21,9	112,6	24,1
	90°	118,2	23,7	114,9	24,2
**3 000 Hz**	**-90°**	**126,2**	**26,0**	**113,3**	**37,6**
	-45°	125,9	20,1	111,5	40,5
	0°	116,1	24,5	113,1	38
	45°	117,8	27,0	117,8	28,4
	90°	113,1	31,1	116,2	30,5
**600 Hz**	**-90°**	**137,5**	**20,2**	**125,6**	**37,1**
	-45°	139,3	22,5	122,3	39,5
	0°	135,4	20,1	130,9	32,5
	45°	126,5	22,5	125,8	28,7
	90°	132,1	24	129,6	30,7

The grand-average amplitude (dipole moment) and peak latency for ECDs calculated for the N1m responses to stimuli with four different bandwidths and presented from five frontal horizontal directions.

Overall, the N1m tended to be larger in amplitude and shorter in latency in the right hemisphere, but the differences did not reach statistical significance due to interindividual variation. Both N1m amplitude (*F*[4,24] = 9.33, *p *< 0.001) and latency (*F*[4,24] = 11.51, *p *< 0.001) exhibited directional tuning, being respectively larger and shorter for contralateral than ipsilateral stimulation. The mean N1m amplitude was affected by the bandwidth of the stimuli, being larger for all stimuli with reduced bandwidth and largest for the stimuli with a bandwidth of 3 kHz. Separate analysis of each hemisphere revealed that while the effects of narrowing the bandwidth on the right-hemispheric N1m amplitude were statistically significant (*F*[3,21] = 10.29, *p *< 0.001), the effects on the left-hemispheric N1m amplitude were not. In both hemispheres, the peak latency of the N1m was, on average, 14 ms longer for the stimuli with a bandwidth of 600 Hz than for the other stimuli (*F*[3,18] = 8.50, *p *< 0.001). The right-hemispheric N1m source location for the 600 Hz stimuli was on average 2 mm more anterior than for the other stimuli (*F*[3,21]) = 5.54, *p *< 0.01). No other significant differences in N1m source location between stimuli were observed.

**Figure 3:**
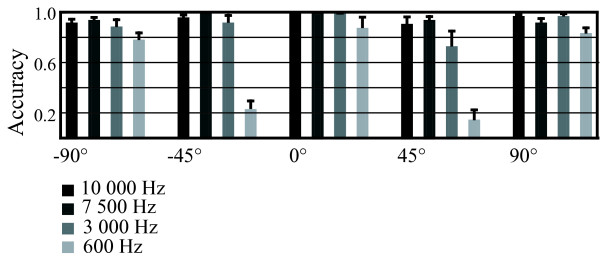
**Direction discrimination accuracy**. The subjects' grand-average accuracy in discrimination of the sound source direction. Stimuli with four different bandwidths were presented from five frontal horizontal directions. Stimuli with the narrowest bandwidth were poorly localized, particularly if originating from the oblique direction angels (-45°, 45°). Error bars indicate SEM.

In the behavioral experiment, subjects made only occasional errors in discriminating the direction of the broadband stimuli. Reducing the bandwidth of the stimuli had no significant effect on the ability of the subjects to discriminate the sound direction until the bandwidth was only 600 Hz (see Fig. 3; *F*[3,21] = 42.76, *p *< 0.001). The narrowest bandwidth made especially the localization of the oblique direction angles (-45° and 45°) very difficult, with performance at chance level (*F *[12,84] = 8.63, *p *< 0.001) and the subjects tending to localize these sounds to their corresponding orthogonal direction (-90° and 90°) instead.

## Discussion

The present study was conducted to investigate the possible contribution of natural spectral information to cortical activity underlying the perception and localization of spatial sounds in the horizontal plane. A previous MEG and behavioral experiment utilizing horizontal spatial sounds, conducted by Palomäki et al. [17], demonstrated that when compared with non-individualized stimuli with isolated ITD and ILD cues, binaurally recorded, individualized wide-band sound stimuli containing direction- and ear-specific spectral modulations elicited a right-hemispheric cortical N1m response with an increased difference in amplitude between contralateral and ipsilateral stimulation. The contra-ipsi difference, or dynamic range, of the right-hemispheric N1m amplitude was furthermore linked to horizontal localization accuracy of the subjects. The previous results thus suggested that although spectral localization cues are known to be most important for vertical localization [1,2], natural spectral information may contribute also to brain activity related to the perception and localization of sounds originating from horizontal directions.

To investigate the issue, the individualized spatial stimuli from the previous experiment were digitally manipulated in order to reduce their bandwidth, eliminating the predominantly high-frequency spectral modulations in a controlled, stepwise manner while preserving the interaural differences the stimuli contained. MEG measurements utilizing the novel stimuli revealed that the reduction in bandwidth had no significant effect on the dynamic range of the right-hemispheric N1m response even in the most extreme case, where all frequencies above 600 Hz were eliminated. The results indicate that the right-hemispheric cortical activity indexed by the N1m dynamic range, linked to horizontal sound localization by Palomäki et al., is insensitive even to a very dramatic reduction in bandwidth and thus the amount of high-frequency spectral information present. Furthermore, the subjects' accuracy in the behavioral direction discrimination experiment was not affected until all frequencies above 600 Hz were removed. It should be noted, however, that some of the behavioral results may have suffered from a ceiling effect (see Fig. 3), which may have prevented potentially subtle differences between the conditions from becoming observable. A direct comparison between the MEG and the behavioral results is also made difficult by the former having been gained using the passive listening condition and the latter using active discrimination.

The present results seem to be very well in accord with behavioral-level psychophysical findings indicating the low relevance of spectral cues to horizontal localization [1,2]. The results of Palomäki et al. [17] show that horizontal stimuli with realistic spectral and interaural information caused a larger N1m dynamic range than stimuli with interaural cues only. In light of the present evidence, it is unlikely that the enlarged dynamic range was elicited in response to the natural spectral structure of the stimuli, unless it is assumed that binaural spectral cues even at frequencies below 600 Hz are sufficient to elicit enhanced cortical activity. The previous results can thus most likely be attributed to other individualized spatial stimulus properties, present at relatively low frequencies and sufficient to elicit enhanced localization-related brain activity. The question then remains as to exactly which properties allowed the auditory system to differentiate between the individualized and non-individualized stimuli. One possible explanation is that while the isolated ITD and ILD cues were carefully constructed to reflect the direction-dependent interaural differences present in the binaurally recorded stimuli, they were averaged over the entire subject population and therefore non-individualized. The observed increase in localization-related brain activity may thus have been due to the more realistic, individualized nature of the interaural cues within the binaurally recorded stimuli. Should this be the case, it would indicate that the auditory areas of the human cortex are surprisingly sensitive even to relatively small details in interaural localization cues present within incoming sound stimuli. Finally, it is worth noting that any conclusions drawn from the present results are valid only for frontal horizontal directions. In the previous study, three of the eight horizontal directions used were located behind the subject, and the subjects made fewer front/back confusion errors when presented with the stimuli with realistic spectral information. It is not inconceivable that the analysis of spectral cues to discriminate between sound stimuli originating from the front and the back of the subject (see [28]) could somehow contribute to brain activity related to horizontal localization.

Unexpectedly, in the present study the absolute amplitude of the right-hemispheric N1m response was found to be larger for stimuli with a reduced bandwidth. In the left hemisphere, this effect was also visible but not statistically significant. A possible explanation for this rise in amplitude is that as high frequencies were removed from the stimuli, sound energy was added to the remaining frequency range to keep the ILD cues constant. Thus, the lower-bandwidth stimuli contained more sound energy in the frequency range to which the human auditory system is especially sensitive (see e.g., [2]). The increased N1m amplitude might therefore reflect the increased input in this frequency range. Even more surprisingly, the dynamic range of the left-hemispheric N1m was found to be sensitive to the decrease in bandwidth (see Fig. 2). All stimuli with a reduced bandwidth elicited a left-hemispheric N1m with a diminished dynamic range. This result might be taken as an indication of left-hemispheric processing of high-frequency sound information, but is unlikely to be related to sound localization, as the right-hemispheric dynamic range and localization accuracy both remained unaffected. It is also noteworthy that in the experiment of Palomäki et al., the availability of localization cues within the stimuli was not reflected in left-hemispheric cortical activity [17].

Even though the removal of high-frequency spectral information from binaural spatial sounds did not affect horizontal direction discrimination and related brain activity in the present experiment, the fact remains that spectral localization cues are most likely to have some function in the perception of any environmental auditory object. While interaural cues are sufficient for accurate discrimination between horizontal sound directions, they alone do not create the subjective perception of the sound originating from outside of the listener's head. On the contrary, binaural spectral information appears to be necessary for the listener to localize a sound source as being external (see e.g., [1,30]). In the present experiment, subjects tended to perceive most of the stimuli with reduced bandwidths as originating from an external sound source. This tentative result is in line with previous results indicating that sound-source externalization depends on low-frequency interaural phase components below approximately 1 kHz and on realistic spectral profiles being present in both ears ([30]; however, see [33] for results suggesting that realistic sound spectrum detail is not necessary for an externalized perception).

The stimuli with the most reduced bandwidth (600 Hz) seem to fall into a class of their own with regard to elicited brain activity and direction discrimination accuracy. Firstly, the N1m responses to these stimuli were considerably delayed in both hemispheres, and the right-hemispheric N1m sources were slightly more anterior than for stimuli with a wider bandwidth. It is questionable, however, whether the longer latency was related to spatial properties; for similarly delayed N1m responses to non-spatial low-frequency stimuli, see [34]. Secondly, these stimuli were localized poorly, particularly when occurring at the oblique direction angles (-45°, 45°), and were subjectively perceived as not originating from an external sound source. While the contribution of ILD cues at such low frequencies may have been diminished [2], the stimuli of the narrowest bandwidth still should have retained usable ITD cues, which are generally sufficient for horizontal localization [25]. The poor localization performance and internalized perception could be due to an error in the reproduction of low-frequency phase information, since no correction was made for the headphone-to-eardrum transfer function in the behavioral experiment utilizing headphones. Furthermore, a response bias has been previously reported, where subjects tend to localize sound sources at oblique angles as shifted towards the corresponding side (see [17]).

Extending on the wealth of psychophysical, neuropsychological and animal model research on sound localization, the cortical activity underlying the human perception and localization of naturally occurring sounds can now be investigated in detail with MEG experiments utilizing natural spatial sound stimuli. Neuromagnetic experiments utilizing realistic spatial stimuli can be especially beneficial for investigating the neural processing of binaural spectral cues, whose contribution to sound localization and integration with the interaural (ITD and ILD) cues is not yet completely understood. In particular, the contribution of high-frequency spectral cues to the discrimination of elevation differences can be seen as a promising subject of such experiments (for an example, see [13]), as spectral cues are known to be essential for elevation discrimination. Interestingly, the sensorineural hearing loss that typically occurs with aging and degrades our ability to process high-frequency sounds [2] can, in turn, lead to deficits in sound localization [35]. Further studies aimed at resolving how the auditory system deals with the presence or absence of high-frequency spectral information could thus also contribute to the understanding of sound localization deficits and high-frequency hearing dysfunction in general.

## Conclusion

The present results indicate that the cortical processes underlying frontal horizontal sound localization, indexed by the dynamic range of the right-hemispheric N1m amplitude, are insensitive to the presence of high-frequency spectral information. The results are thus in accord with previous psychophysical findings on human localization of sounds in the horizontal plane. The previously described changes in localization-related brain activity, reflected in the enlarged N1m dynamic range elicited by natural spatial stimuli, can most likely be attributed to the processing of individualized spatial cues present already at relatively low frequencies. Interestingly, the dynamic range of the left-hemispheric N1m was affected by the removal of high frequencies over 7.5 kHz, perhaps suggesting left-hemispheric processing of high-frequency sound information not related to localization. Taken together, these results also provide converging evidence for a hemispheric asymmetry in sound localization.

## Methods

### Subjects

Eight volunteers (right-handed, mean age 40 years, two females) served as subjects with informed consent in the experiment, which was approved by the Ethical Committee of Helsinki University Central Hospital. The subjects participated in an MEG measurement and in a subsequent behavioral listening experiment. All subjects had also participated in the previous MEG study [17].

### Stimulus preparation and presentation

The stimulus set comprised bursts of uniformly distributed white noise with four different bandwidths presented from one of five frontal horizontal directions (-90°, -45°, 0°, 45°, 90°; angle 0° in front of the subject, angles >0° in the right frontal hemifield). The stimuli used in the previous experiment [27] were used as base stimuli. Broadband (0–10 kHz) noise bursts were used to ensure the generation of spectral cues over a wide bandwidth. The stimuli were recorded binaurally and individually for each subject. The recordings of 200-ms epochs took place in a slightly reverbant room adhering to the IEC-268-13 standard for listening rooms [36]. Miniature microphones were attached to the entrance of the subject's ear canals, which were blocked using silicone paste, and 50-ms noise bursts were sequentially presented through loudspeakers located at a distance of 1.5 m in the five horizontal directions. For the purposes of the present study, the bandwidth of the recorded noise sequences was narrowed using zero-phase low-pass finite impulse response (FIR) filters. The cut-off frequencies of the filters were adjusted in the Bark scale as follows: 10 000 Hz (100% of original bandwidth); 7 500 Hz (90%); 3 000 Hz (67%); 600 Hz (33%). ITDs and ILDs were, importantly, left intact in the filtering. For the ITD cues, this was enabled by the use of zero-phase FIRs. To avoid changing the ILD cues, the sound energy lost in the low-pass filtering process was compensated for by adding exactly the same amount of energy equally over the remaining frequency range. In both the MEG and the behavioral experiments, the stimuli were presented to the subjects at at 75 dB A-weighted Sound Pressure Level (SPL) measured for the 10 kHz stimulus from direction 0°.

During the MEG experiment, the subject sat in a reclining chair under instruction not to pay attention to the auditory stimuli and to concentrate on watching a self-selected silent video. The stimuli were then presented through a custom-made wide band tubephone sound system (frequency range 100 Hz – 10 kHz [37]; the same sound system was used in the previous MEG experiment [17]). Blocks of approximately 150 instances of one stimulus type (i.e., combination of direction and filter level) were presented sequentially in counterbalanced order at an onset-to-onset stimulus interval of 1500 ms. In the behavioral experiment, the same sound stimuli were presented to the subject through high precision headphones in a sound-proof room. The stimuli were organized in randomized blocks, each containing 15 stimuli with the same bandwidth presented from 5 different directions. Each stimulus type was presented 12 times in total. The subject's task was to indicate the direction of each sound presented by using a computer keyboard.

### Data acquisition and analysis

Brain activity was recorded (passband 0.01–200 Hz, sampling rate 1000 Hz) with a 306-channel whole-head MEG device (Vectorview 4-D, Neuromag Oy, Finland). Before the acquisition, the dimensions of the skull and the location of the head position indicator (HPI) coils were measured using a 3-D digitizer, and HPI coil positions were determined prior to the presentation of each stimulus block. Horizontal and vertical eye movement sensors were used for removing epochs with eye movement artefacts defined as absolute displacements exceeding 150 μV. Over 150 responses for each stimulus type were collected for each subject and averaged in order to cancel out the cortical activity not time-locked to stimulus presentation. Averaged responses were baseline-corrected with respect to a 100-ms pre-stimulus baseline and band-pass filtered at 1–20 Hz.

Auditory N1m responses, defined as the peak of the negative deflection in the individual event-related response beginning at around 100 ms, were characterized for each subject through equivalent current dipoles (ECDs) [38]. Separate subsets of 44 gradiometer sensors over the temporal areas of the left and right hemispheres were used in the ECD estimation. The head-based coordinate system was defined by the *x*-axis passing through the preauricular points (positive to the right), the *y*-axis passing through the nasion, and the *z*-axis as the vector cross product of the *x- *and *y-*unit vectors. The average goodness-of-fit of ECDs for all stimuli was 91%, with the minimal accepted goodness-of-fit being 60%. The left-hemispheric data for one subject was discarded due to too low goodness-of-fit values. In addition to the ECD properties (moment/amplitude, latency, location) the dynamic range of the N1m response was investigated for each subject and stimulus bandwidth by subtracting the mean N1m amplitude for ipsilateral hemifield stimuli from its contralateral counterpart. In the left hemisphere, the mean of the N1m amplitudes evoked by the left-hemifield (-90°, -45°) stimulation was subtracted from the mean of the N1m amplitudes evoked by the right-hemifield (90°, 45°) stimulation, while in the right hemisphere, the mean N1m amplitude evoked by right-hemifield stimulation was subtracted from the mean N1m amplitude evoked by left-hemifield stimulation. The procedure used to calculate the N1m dynamic range replicated the procedure in the previous experiment [17] and is based on the findings of numerous studies that for both hemispheres, activation in the auditory cortex is maximal for contralateral and minimal for ipsilateral stimulation (e.g., [13,15,16]).

Differences according to stimulus type in the N1m amplitude, latency, location and dynamic range as well as in accuracy in the listening experiment were statistically investigated with *M*-way repeated measures analyses of variance (ANOVAs) and Newman-Keuls post-hoc comparisons.

## Competing interests

The author(s) declares that there are no competing interests

## Authors' contributions

PA, PM and HT designed the experimental setup of the study, and PA prepared the auditory stimuli. SL and LL acquired the data. SL performed the data and statistical analyses and prepared the manuscript. All authors participated in the writing process, and have approved the final version of the manuscript.
